# Correlation between congenital bicuspid aortic valve morphology with thoracic aortic dimensions - a retrospective analysis with cardiovascular magnetic resonance

**DOI:** 10.1186/1532-429X-15-S1-P292

**Published:** 2013-01-30

**Authors:** Lay Koon Tan, Beatrice Bonello, Yumi Shiina, Raad H Mohiaddin

**Affiliations:** 1 CMR, Royal Brompton Hospital, London, UK; 2 National Heart and Lung Institute, Imperial College, London, UK; 3 Cardiology, National Heart Institute, Kuala Lumpur, Malaysia

## Background

Congenital bicuspid aortic valve (BAV) is the commonest inherited cardiac defect that is often associated with a medley of other cardiovascular anomaly, the most frequent being aortic dilatation which may be a consequent of an interplay between genetic and hemodynamic factors. This retrospective study aims to find a correlation between BAV valve morphology with thoracic aortic dimension with cardiovascular magenetic resonance imaging (CMR).

## Methods

A retrospective analysis of the aortic valve and aortic (Ao) dimension was made of 149 patients aged between 6 and 77 years (mean age 43±17 ,102 males and 47 females) with BAV who underwent CMR study. BAV patients with associated coarctation or valvular dysfunction were excluded. Images were acquired with either a Siemens Avanto or Sonata 1.5T scanner in orthogonal planes. Aortic maximal intraluminal dimensions in end diastole were measured at the annulus, sinuses, sino-tubular junction, mid ascending aorta, arch and proximal descending aorta. These measurements were indexed according to body surface area (BSA). BAV valve morphology were characterised in cross-sectional steady-state free precession (SSFP) cine images and grouped according to the presence or absence of a raphe.

## Results

Of the valve morphology, majority of patients (59.7%) had a raphe, almost always seen between the fused right and left cusps (89%). The indexed aortic root and ascending aortic dimensions were found to be smaller in patients with remnant raphe BAV as compared with those without a raphe (‘pure' BAV). There was a trend towards smaller indexed proximal aortic arch dimensions in BAV patients with valvular raphe although this did not reach statistical significance (p=0.07). There were no difference in indexed aortic arch and proximal descending aortic dimension between the 2 groups as shown in the table below:

**Table 1:** Median Indexed Aortic Dimension in BAV with raphe and Pure BAV patients

	BAV with raphe Median(95%CI) (mm/m2)	Pure BAV Median(95%CI) (mm/m2)	Significance (p value)
Aortic Annulus	14.0 (13.7-14.5)	14.9 (14.3-15.5)	0.007
Aortic Sinus	18.2 (17.7-18.9)	20.7 (19.9-21.7)	0.0001
Sinotubular Junction	14.2 (13.7-14.6)	16.1 (14.5-17)	0.004
Mid Ascending Aorta	17.8 (17.2-18.6)	19.2 (18.3-20.4)	0.03
Proximal Arch	13.5 (13-14.3)	14.0 (13.2-14.9)	0.07
Mid Arch	11.0 (10.7-11.5)	11.3 (10.6-11.9)	0.7
Proximal Descending Aorta	10.6 (10.1-10.9)	10.5 (10-11.1)	0.9

CI - confidence interval

## Conclusions

Majority of BAV patients have a remnant raphe, commonly between the fused right and left cusps. These patients were also found to have smaller indexed aortic root and ascending aortic dimensions compared with those with ‘pure' BAV which may indicate an inherent difference in biologic aortic structure.

## Funding

No funding

